# The 2022 West Nile Virus Season in Greece; A Quite Intense Season

**DOI:** 10.3390/v15071481

**Published:** 2023-06-29

**Authors:** Danai Pervanidou, Chrysovaladou Niki Kefaloudi, Anna Vakali, Ourania Tsakalidou, Myrsini Karatheodorou, Katerina Tsioka, Maria Evangelidou, Kassiani Mellou, Styliani Pappa, Konstantina Stoikou, Vasiliki Bakaloudi, George Koliopoulos, Kostas Stamoulis, Eleni Patsoula, Constantina Politis, Christos Hadjichristodoulou, Anna Papa

**Affiliations:** 1 National Public Health Organization (EODY), 151 23 Athens, Greece; 2 National Reference Center for Arboviruses and Haemorrhagic Fever Viruses, Department of Microbiology, Medical School, Faculty of Health Sciences, Aristotle University of Thessaloniki, 541 24 Thessaloniki, Greece; 3 Hellenic Pasteur Institute, 115 21 Athens, Greece; 4 Molecular Blood Center, AHEPA University General Hospital, 546 36 Thessaloniki, Greece; 5 Department of Crop Science, School of Plant Sciences, Agricultural University of Athens, 118 55 Athens, Greece; 6 Hellenic National Blood Transfusion Center, 136 72 Athens, Greece; 7 Department of Public Health Policy, School of Public Health, University of West Attica, 115 21 Athens, Greece; 8 Department of Hygiene and Epidemiology, School of Health Sciences, Faculty of Medicine, University of Thessaly, 412 22 Larisa, Greece

**Keywords:** West Nile virus, Greece, mosquito-borne disease, vector-borne disease

## Abstract

Since 2010, the West Nile virus (WNV) has been established in Greece. We describe the epidemiology of diagnosed human WNV infections in Greece with a focus on the 2022 season. During the transmission period, clinicians were sending samples from suspected cases for testing. Active laboratory-based surveillance was performed with immediate notification of diagnosed cases. We collected clinical information and interviewed patients on a timely basis to identify their place of exposure. Besides serological and molecular diagnostic methods, next-generation sequencing was also performed. In 2022, 286 cases of WNV infection were diagnosed, including 278 symptomatic cases and 184 (64%) cases with neuroinvasive disease (WNND); 33 patients died. This was the third most intense season concerning the number of WNND cases, following 2018 and 2010. Most (96%) cases were recorded in two regions, in northern and central Greece. The virus strain was a variant of previous years, clustering into the Central European subclade of WNV lineage 2. The 2022 WNV season was quite intense in Greece. The prompt diagnosis and investigation of cases are considered pivotal for the timely response, while the availability of whole genome sequences enables studies on the molecular epidemiology of the disease.

## 1. Introduction

West Nile virus (WNV) is a flavivirus circulating in nature in an enzootic cycle between mosquito vectors and birds [1,2]. Humans and equids are infected through mosquito bites and are considered dead-end hosts [3,4,5]. Transmission through substances of human origin (SoHO) can occasionally occur (mainly through transfusion of blood, blood components, or organ transplantation), indicating the need for safety measures to minimize this additional WNV transmission risk [1,4,6,7,8]. 

While most human infections remain either asymptomatic (approximately 80%) or manifest a mild disease (20%) known as West Nile fever (WNF), less than 1% develop neuroinvasive disease (WNND), such as encephalitis, meningitis, or myelitis/acute flaccid paralysis (AFP) [9,10,11,12]. Advanced age, chronic underlying diseases, and immunosuppression are risk factors for developing severe disease [1,2,13]. WNV lineages 1 and 2 have been associated with disease in humans [1].

WNV has been established in parts of southern, central, eastern, and western Europe, with annual seasonal outbreaks during summer and early autumn [14]. Since at least the late 1950s, strains of WNV lineage 1 have been circulating in Europe and in the Mediterranean basin, causing human outbreaks^,^ [15,16], while WNV lineage 2 was first detected in 2004 in Hungary and then spread and became established in central Europe and the Mediterranean basin, causing major outbreaks [17,18,19,20,21,22,23,24,25]. 

Since 2010, Greece has had one of the highest notification rates in Europe [25]. After the first outbreak in 2010, in the Central Macedonia Region [26,27], the virus further spread to the south and then to the northeast part of the country, with human cases recorded on an almost annual basis [28,29,30,31,32]. Cases have been recorded in all 13 Regions, with almost half (53%) of the regional units (NUTS3) and half (46%) of the municipalities being affected at least once during 2010–2021 [28]. Specific areas, mainly in the northern and central mainland, are considered WNV “hot spots” as they are “affected” during several transmission periods. 

All (except one) WNV sequences detected in patients, blood donors, mosquitoes, horses, and birds from Greece cluster into the Central European subclade of WNV lineage 2 [21,31,33,34,35,36,37,38,39,40,41,42,43,44,45,46,47,48]; one sequence of the Eastern European/Russian subclade of WNV lineage 2 was detected in a human case in northeastern Greece in 2018, indicating the introduction of a new virus [49].

Since 2010, enhanced surveillance of human WNV infection has been implemented by the National Public Health Organization (NPHO), especially from May to November, in order to promptly detect human cases, identify their temporal and geographical distribution, and inform and guide the relevant national, regional and local authorities to implement targeted response measures promptly, i.e., blood safety measures, intensified vector control, and communication campaigns. 

In this study, we describe the epidemiology of diagnosed human WNV infections in Greece with a focus on 2022, comparing their distribution in time and place with that in previous years.

## 2. Methods

### 2.1. Surveillance and Case Investigation—Data Collection

WNV is a mandatory notifiable disease in Greece, and all laboratory-diagnosed cases of WNV infection, symptomatic or not, with or without neuroinvasive disease, should be notified to the NPHO.

The EU case definition of WNV infection [50] was used with slight modifications (in that (i) only laboratory—and not epidemiological—criteria were used to define probable cases, and (ii) PCR detection of WNV nucleic acid in urine was also used for case confirmation). Probable cases (i.e., cases with a positive IgM antibody response only in serum) were also included in the database and analysis. Since 2018, infections (asymptomatic or not) among blood donors detected during blood screening were also included in the database. 

In May 2022, in the context of routine enhanced WNV surveillance in humans, an information and awareness letter was sent to all health units and medical associations in the country for vigilance regarding WNV, recommending testing for WNV infection of suspected cases. Following the recording of cases in an area, local health units were urgently informed to enhance their vigilance.

NPHO recommended the referral of samples to the National Reference Centre (NRC) and other specialized laboratories. The Vector-borne Diseases (VBD) Department of the NPHO ran active laboratory-based surveillance, throughout the transmission season, with daily communication and information exchange with the laboratories, which notified the diagnosed cases to NPHO on a daily basis. In addition, blood transfusion services informed the Coordinating Hemovigilance Centre and Surveillance of Transfusion (SKAEM) of NPHO about WNV infection cases among blood donors.

The VBD Department investigated every diagnosed WNV case with in-depth telephone interviews with the treating physicians and the patients or their close relatives, ideally within 24–48 h after diagnosis, using a standardized investigation form. The case investigation aimed to (i) determine the probable place of exposure (according to the travel history during the maximum incubation period), (ii) detect risk factors for WNV infection and increased severity of the disease (including underlying diseases, outdoor activities, occupation, mosquito nuisance and nearby large mosquito breeding sites, and recent blood transfusion or organ transplantation) and (iii) record the clinical manifestations and severity of the disease (the symptoms and the clinical form, i.e., WNND or WNF, based on the treating physicians’ clinical assessment and the laboratory/imaging findings, when available). The health status of hospitalized cases was actively updated on a daily basis to record the final outcome. Positive blood donors were investigated by the relevant blood transfusion services, in accordance with protocols of SKAEM, and by the NPHO. Weekly surveillance reports were published on the NPHO website.

A daily update on the diagnosed cases of all relevant national, regional, and local stakeholders was performed by the NPHO, in order to guide targeted response measures (blood safety, vector control, communication).

### 2.2. Designation of Areas Affected by WNV

Each municipality where at least one human case of WNV infection (either with WNND or not) was exposed during the current transmission season was defined as “affected” by WNV. Lists of the affected municipalities were published on the NPHO website. The designation of affected municipalities was conducted by a multi-sectoral “Working Group (WG) for the designation of areas affected by VBDs” of the Ministry of Health. In case of a complex travel history during the incubation period, this WG was immediately notified by the NPHO and consulted on the most probable place of exposure, after assessing all available recent and historical epidemiological and entomological information. 

Throughout the country, blood safety and hemovigilance measures were implemented for blood donors residing or having visited affected municipalities (either screening of donated blood for WNV RNA, with targeted individual donation (ID) nucleic acid amplification testing (NAT), or blood donor deferral, and hemovigilance including post-donation and post-transfusion information) [51,52,53]. 

### 2.3. Laboratory Methods

Serum and cerebrospinal fluid (CSF) specimens were tested for the presence of WNV-specific IgM and IgG antibodies using the commercial ELISA kits WNV IgM capture DxSelect and WNV IgG DxSelect (Focus Diagnostics Inc., Cypress, CA, USA) following the instructions of the manufacturers; an index >1.1 for IgM and >1.5 for IgG was defined as a positive result. For the molecular methods, RNA was extracted from patients’ blood, CSF, or urine samples using the QIAamp Viral RNA Mini kit (Qiagen, Hilden, Germany). WNV RNA amplification was performed using either the commercial RealStar WNV *RT-PCR* Kit 2.0 (Altona Diagnostics, Hamburg, Germany) or the in-house real-time RT-PCR protocol described by Linke et al. [54]. Known WNV-positive RNAs were used as positive controls. In addition, the NRC performed an RT-nested PCR [35] combined with Sanger sequencing, as well as next-generation sequencing (NGS) using a recently designed PCR-based protocol, on samples that showed a cycle threshold (Ct) of less than 30 in the real-time RT-PCR in order to obtain whole genome sequences [55]. NGS was performed on an Ion PGM sequencer, using a 316 Chip. Assembly and annotation were conducted in Geneious Prime, version 2021.2.1. The sequence of the Nea Santa-Greece-2010 strain (HQ537483) was used as a reference. A maximum likelihood phylogenetic tree was constructed using MEGA version 11 software [56]. 

### 2.4. Data Analysis

A descriptive analysis of the surveillance data was performed, concerning the geographical and temporal distribution of human cases with WNV infection, their demographic characteristics (age, sex), clinical manifestations, underlying diseases, and clinical outcome. A comparison with previous transmission seasons was also performed, regarding the number and geographical and temporal distribution of cases, with a particular focus on the two most intense 2010 and 2018 seasons. Week numbers were assigned using the International Organization for Standardization (ISO) 8601 standard [57].

### 2.5. Ethical Statement

No ethical approval was needed for this study, as only aggregated data were analyzed and presented without any identifiable data.

## 3. Results

**Figure 1:**
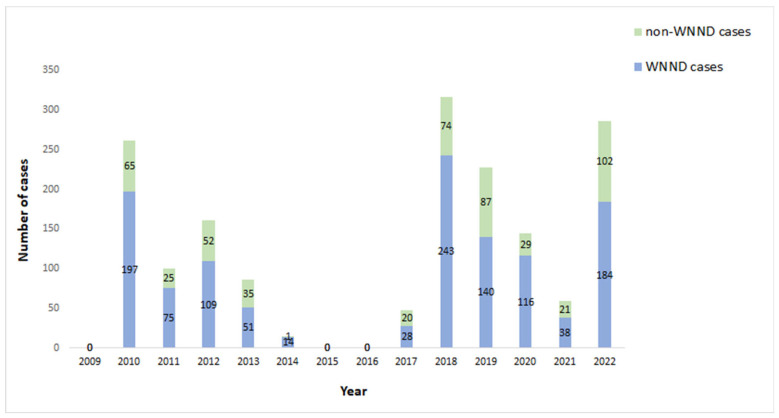
Number of WNND and non-WNND ∗ cases by year, Greece, 2010–2022. Both West Nile fever (WNF) and asymptomatic cases are included in the non-WNND cases.

In the 2022 period, 286 laboratory-diagnosed cases of WNV infection were reported to NPHO; 278 cases were symptomatic, and 184 of them presented with WNND. Among the 102 non-WNND cases, 94 presented mild symptoms (WNF), and 8 were asymptomatic blood donors (Table 1). A total of 19 blood donors were diagnosed through blood screening in affected areas (age 18–59 years); 11 of them developed mild symptoms and 8 remained asymptomatic. The overall WNND notification rate was 1.7 cases per 100,000 population; this was the third-highest notification rate recorded in Greece, following the 2018 and 2010 seasons (2.2 and 1.8 cases per 100,000 population, respectively) (Table 1, Figure 1). 

Hemovigilance procedures demonstrated that one WNND case acquired the infection through blood transfusion; the patient recovered. The implicated donation was made before preventive measures had been triggered in this municipality.

Regarding the timeliness of case investigation, for 264 cases investigated by the VBD Department of the NPHO (excluding the 19 blood donors and cases with non-available relevant information), the median period from diagnosis to case investigation was one day (range: 0–5). From July to October 2022, the “WG for the designation of areas affected by VBDs” assessed and consulted on the most probable place of exposure for 55 cases with complex travel history during the incubation period.

**Figure 2:**
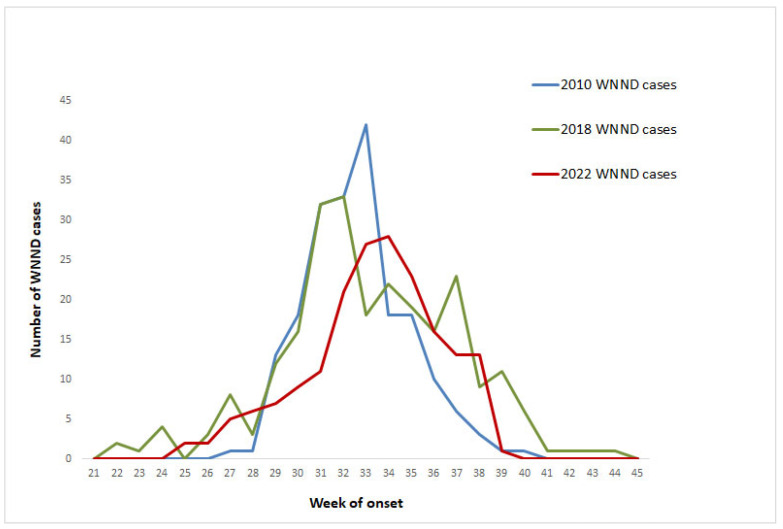
Number of WNND cases by week of symptom onset in 2022, 2018, and 2010 (the three most intense WNV seasons), Greece.

The first diagnosed case of WNV infection in 2022 (case with WNND) was diagnosed and reported on 7 July and had an onset of symptoms on 23 June (week 25/2022), and the last diagnosed case (asymptomatic blood donor) had blood sampling (donation) on 20 October (week 42/2022). The number of recorded WNND cases peaked in weeks 33 and 34, later than in the previous two most intense seasons (2018 and 2010) (Figure 2). In 2010 and 2018, almost half of WNND cases had occurred up to week 32 (approximately mid-August) (50% and 47%, respectively), in contrast to 2022 (when 34% of WNND cases had occurred up to week 32). In addition, in 2022, a total number of 87 (30%) cases had symptom onset or sampling in September–October, including 61 (33%) WNND cases. The number of WNND cases with symptom onset up to the end of July (n = 31) was similar to the number of cases in the same season of the five previous years with the highest annual number of cases, i.e., 2010 (n = 28), 2012 (n = 36), 2018 (n = 55), and 2019 (n = 28). Figure 2 shows the reported WNND cases by week of symptom onset in 2022, compared with the two most intense previous seasons (2010 and 2018).

In all except two cases, the probable place of exposure could be determined. For cases with available information on their probable place of exposure, 83% (232/282) were considered infected in their place of permanent residence, while the rest were considered in their place of summer vacation. 

**Figure 3:**
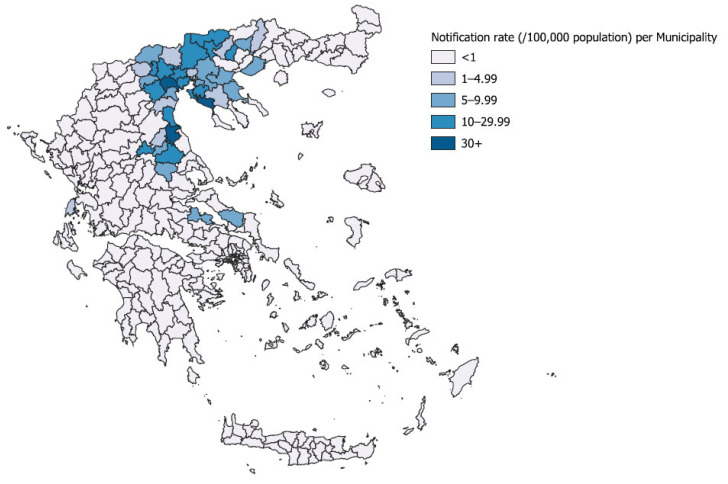
Notification rate (per 100,000 population) of WNND by probable municipality of exposure and geographical distribution of WNND cases, Greece, 2022 (n = 183).

The cases were recorded in 46 (14%) municipalities, 14 (19%) regional units, and 5 (38%) regions of Greece [Central Macedonia, Thessaly, East Macedonia and Thrace, Central Greece (Sterea Ellada), and the Ionian islands]; the geographical distribution of cases was relatively limited compared to 2018 (Table 1). The regions and all regional units affected in 2022 were affected also in previous transmission seasons. At the municipality level, cases were recorded for the first time in three new municipalities. The geographical distribution of WNND cases by municipality of probable exposure is presented in Figure 3. 

**Figure 4:**
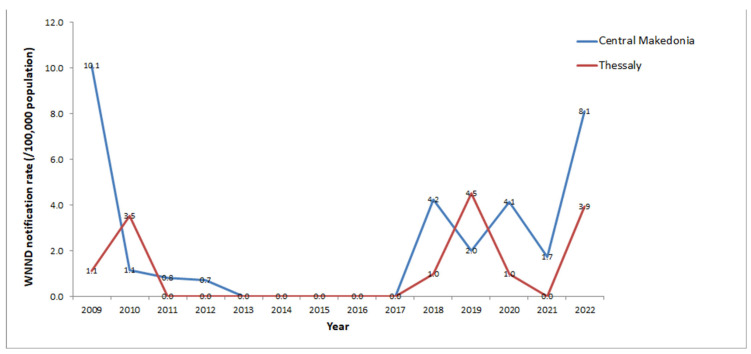
Annual notification rate (number of cases/100,000 population) of WNND in Central Macedonia and Thessaly Regions, 2010–2022. Calculations based on the 2011 and 2021 census data [58].

The vast majority (96%) of WNND cases in 2022 were recorded in two regions (81% in Central Macedonia and 15% in Thessaly), representing two major epicenters with high local notification rates. The second highest WNND notification rates ever were recorded in both regions (Figure 4); 8 WNND cases/100,000 population in Central Macedonia (the second largest following the 2010 season), and 4 WNND cases/100,000 population in Thessaly Region (the second largest after 2019) (Figure 4). The highest notification rates since 2010 were recorded in three regional units and 19 municipalities (14 in Central Macedonia and four in Thessaly Region). Both urban areas, including large cities/capitals of regional units, and rural areas were affected.

**Figure 5:**
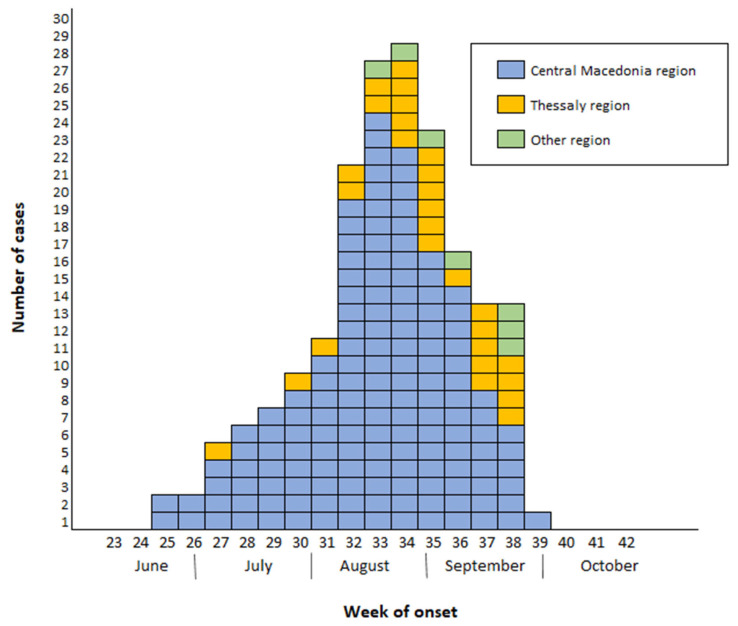
Number of laboratory-diagnosed West Nile virus neuroinvasive disease (WNND) cases by week of symptom onset and region of exposure, Greece, 2022 (n = 286).

The first three cases of 2022 (all with WNND) had symptom onset in late June and were recorded in urban areas with high population density in the Central Macedonia Region. In July, cases occurred also in Thessaly Region (Figure 5). From August to October, these regions continued to be the major epicenters, whereas from late August eight sporadic cases were recorded in three additional regions.

The median age of WNND cases was 76 years (range: 14–96 years). Among the 184 WNND cases, 91% (n = 169) were aged ≥50 years, 65% (n = 119) were aged ≥70 years, and 36% (n = 66) ≥80 years. The notification rate of WNND among cases aged ≥70 years was 10.5 times higher than that among cases < 70 years (95% CI = 7.8–14.3, *p* < 0.001). The notification rate of WNND cases increased from 0.2 per 100,000 population aged <50 years to 11.3 per 100,000 population aged ≥80 years (RR = 50.5, 95%CI = 28.8–88.4, *p* < 0.001, calculations based on 2011 census data from the Hellenic Statistical Authority [58]).

More than half of the total cases (58%; 165) and WNND cases (56%; 103) were males. The WNND notification rate was 2.0 cases/100,000 population among males, and 1.5 cases/100,000 population among females (calculations based on 2021 census data from the Hellenic Statistical Authority [58]). The WNND incidence among males was 1.3 times higher than that among females (*p* = 0.05).

The median period from symptom onset to diagnosis, for 265 cases with available information (and excluding the cases among blood donors), was 10 days (range: 2–37 days). The median period from hospital admission to diagnosis for 243 hospitalized cases with available information (and excluding the cases among blood donors) was 5 days (range: 0–20). 

Most WNND cases (127/184, 69%) presented symptoms of encephalitis, while 27% (49 cases) presented symptoms of meningoencephalitis, and 4% (8 cases) presented symptoms of meningitis. Nine patients also presented AFP (along with encephalitis or meningoencephalitis). 

A total of 278 (97%) cases reported clinical symptoms, while eight cases (blood donors) remained asymptomatic. Among WNND cases, 28% presented extrapyramidal signs (tremor, Parkinsonism), 21% ataxia/gait disorders, and 15% limp paralysis. A rash was presented in 16% of WNND cases and 29% of WNF cases. Gastrointestinal symptoms (one or more of the following: vomiting, nausea, diarrhea, abdominal pain) were present in 66% and 45% of WNND and WNF cases, respectively.

Among the WNND cases with relevant available information, 86% reported at least one underlying chronic disease; including cardiovascular diseases (72% including stroke and heart disease), heart disease (69%, including hypertension), hypertension (57%), diabetes mellitus (32%), chronic neuropsychiatric disease (9%), respiratory disease (8%), cancer/history of cancer (8%), other immunosuppression than diabetes, chronic renal failure or cancer (7%, including autoimmune diseases and organ transplant), stroke (6%), and chronic renal failure (4%). Among the symptomatic WNF cases, 60% reported at least one underlying chronic disease.

Among the 278 symptomatic cases, 247 (89%) were hospitalized: all 184 WNND cases and 63/94 (67%) symptomatic WNF cases. The median duration of hospitalization of 148 WNND cases (hospitalized and discharged from the hospital) was 8 days (range: 1–103), while among 62 WNF hospitalized cases it was 6 days (range: 1–61). Twenty-seven (10%) out of the 278 symptomatic patients diagnosed with WNV infection in 2022 were hospitalized in an intensive care unit. Thirty-nine (39) patients were not hospitalized.

Thirty-three (33) deaths were recorded during hospitalization, with an overall case fatality (CF) of 12% among all symptomatic cases and 18% among WNND cases (Table 1). Fatal cases concerned patients older than 58 years (median age 83 years). Five fatal cases under 70 years old had underlying diseases (four had severe immunosuppression and one had insulin-dependent diabetes). Three more deaths were attributed to other main causes (and were not included in the total number of deaths among patients with WNV infection). The median period from symptom onset to death (during hospitalization) was 13 days (range: 4–103).

Among cases with available information, 37% (103/279) reported undertaking agricultural or gardening activities, and 13% (36/276) reported having a routine outdoor activity after dawn.

### Laboratory Results

Of the 286 cases, 172 (60.1%) were confirmed either by detection of WNV-specific IgM antibodies in CSF (104; 36.4%) and/or detection of WNV RNA in blood (92; 32.2%), CSF (4; 1.4%), urine (1; 0.3%), and/or in serum (1; 0.3%). A total of 114 cases were considered probable (diagnosis was based only on the detection of WNV-specific IgM antibodies in serum). The median time from symptom onset to sampling for the cases with positive PCR in blood or serum was 5 days (range: −6 to 20).

**Figure 6:**
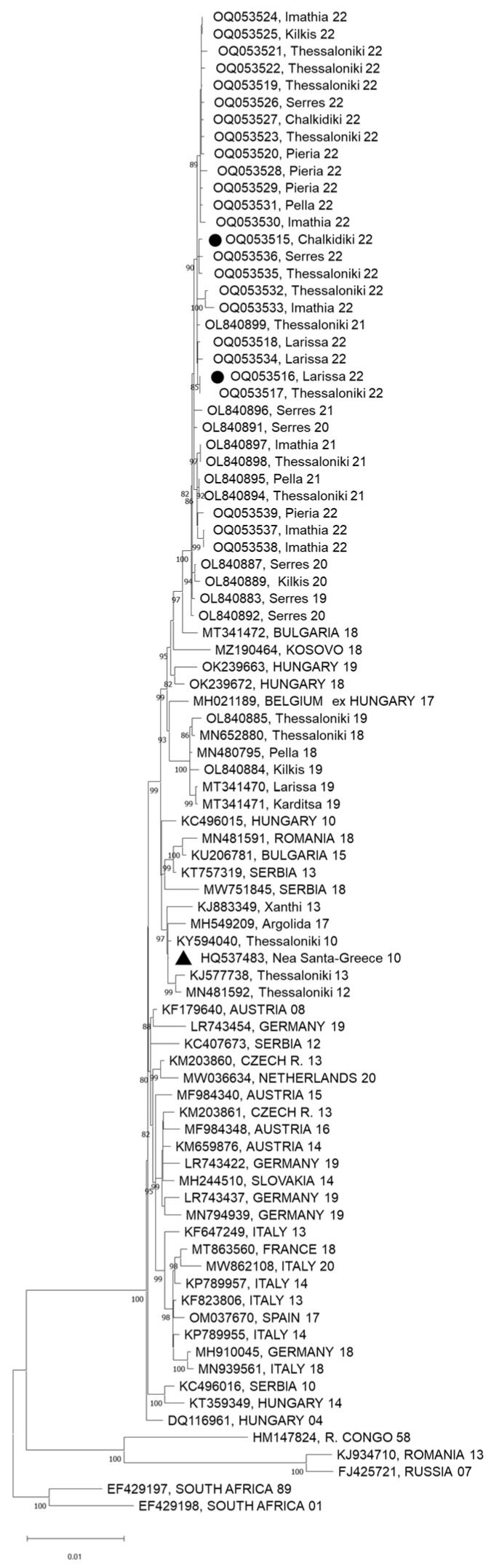
Maximum likelihood phylogenetic tree based on complete nucleotide sequences of WNV lineage 2 polyprotein (10,302 nt). The bootstrap values are shown next to the branches. Sequences of the present study are marked with a circle; the first Greek sequence (HQ537483) is marked with a triangle. The name of the countries is shown in capital letters; all other sequences are from Greece.

The sequences clustered into the Central European/Hungarian subclade of WNV lineage 2, and specifically into a cluster containing all recent Greek sequences taken during 2020–2022 (Figure 6).

## 4. Discussion

Since the first WNV outbreak in 2010, Greece has been one of the most “affected” countries in Europe regarding the annual number and notification rate of cases [25]. In 2018, the highest number of cases was recorded in Europe [25], with large outbreaks occurring in many central and southern European countries. In Greece, the highest number of cases was also recorded in 2018, with the longest transmission season and the widest geographical distribution of cases recorded since the emergence of the virus in the country in 2010 [24].

In 2022, a quite intense circulation of WNV was recorded in many European countries [59,60]. Greece had the second highest notification rate of cases in EU/EEA and EU-neighboring countries, following Serbia, and the second highest number of total cases, following Italy [60].

The high notification rate of WNV disease, placing Greece among the most “affected” countries in Europe in almost each transmission season, is a result of a combination of factors; the location of Greece at the crossing point of migratory bird routes between three continents, the plethora of wetlands, rice fields, and mosquito breeding sites, along with the climate of the country, may favor WNV circulation. Furthermore, the high vigilance of healthcare professionals toward WNV diagnosis and the enhanced WNV surveillance in humans are considered to further contribute to the recording of increased numbers of cases.

The CF among total cases and WNND cases (12% and 18%, respectively), representing the—actively recorded—final outcome of all hospitalized patients, was comparable with the overall CF of total and WNND cases during the period 2010–2021 (15% and 19%, respectively). 

The year 2022 had the third-highest annual WNND notification rate in Greece, following 2018 and 2010 (Table 1). The symptom onset of the first cases in 2022 was in late June. Cases with symptom onset before July occurred in four previous years (2012, 2017, 2018, 2019); in three of these years, an intense WNV circulation was recorded (with an annual number of WNND cases exceeding the median annual number of cases during 2010–2022). The number of WNND cases with symptom onset up to the end of July 2022 was comparable with the median annual number of cases (during the same season) of the five previous most “intense” years (i.e., with the highest annual numbers of cases). Thus, in July 2022, a relatively large number of cases were expected to occur during the current season, in accordance with the previous “intense” years. Additionally, the geographical distribution of the first cases at the onset of the season in large urban areas and cities with high population densities indicated the expected intensity of the season. 

In 2022, cases peaked in late August, 1–2 weeks later than in 2010 and 2018; in early and mid-September an increased number of cases continued to be diagnosed, triggering the publication of a 3rd press release from the NPHO to inform the public about the ongoing intense seasonal outbreak. More than one-third of the cases in 2022 occurred in September and October. 

The geographical distribution of cases in 2022 was quite similar to that in 2010, with epicenters in Central Macedonia and Thessaly Regions. However, in 2022, both rural and urban areas were affected including large cities, in contrast to previous outbreaks in these regions, which occurred mainly in rural areas. 

One major difference between 2022 and 2018 was the geographical distribution of cases and the number of affected areas in the country. The widely spread distribution of cases in 2018, with more than one-third of the regional units affected, indicated a nationwide WNV distribution; in contrast, in 2022, the geographical distribution of cases was more limited. This large number of cases in a rather limited geographical area represented an increased WNV transmission at the local level resulting in high local notification rates. Indeed, the highest WNND notification rates ever were recorded in a total of 19 municipalities and in three regional units. 

In 2022, cases were recorded in 14 regional units where human cases were recorded also in previous years, indicating overwintering and local circulation of the virus and that —once established—the likelihood of maintenance and the risk of re-emergence of WNV infections in the affected areas are high [61]. Three municipalities were affected for the first time. 

From 2010 up to 2022, Central Macedonia was the most “affected” region, with a mean annual notification rate of 2.6 WNND cases/100,000 population, followed by the East Macedonia and Thrace Regions (2.1 WNND cases/100,000 population), and Thessaly Region (1.2 WNND cases/100,000 population). In 2022, the WNND notification rate in Central Macedonia was 8.1 cases/100,000 population, the second highest after 2010 (10.1 WNND cases/100,000 population). 

The Central Macedonia Region has major wetlands (river deltas) and large rice-cultivated areas. These broader areas were the major WNV epicenter in 2010, with 186 WNND cases, the highest number of WNND cases in the region so far. As depicted in Figure 3, fewer cases were reported in the following years, up to 2022, when the second highest number of WNND cases (n = 149) was recorded in the region. The first cases occurred in urban areas/large cities, while later both urban and rural areas were affected. Similarly in Thessaly, rural and urban areas, including large cities, were affected in 2022, with the WNND notification rate slightly lower than the record of 2019 (3.9 versus 4.5 cases/100,000 population). 

Climatic conditions (warm and dry spring, warm and very rainy early summer, with local flooding, especially in the north and central mainland, in the epicenters’ regions) may have contributed to the intense 2022 WNV circulation, as was also suggested for 2018 [47,62]. 

As in previous WNV seasons, WNV sequences in 2022 clustered into the Central European subclade of WNV lineage 2, being variants of a strain introduced in the country (in the Serres Regional Unit) in 2019 [47]. 

The sensitivity of the human surveillance system in Greece is high; enhanced surveillance has been consistently implemented in the country during each transmission season since 2010, including raising awareness among physicians on an annual basis, publishing updated weekly surveillance reports, and providing free-of-charge diagnoses. The enhanced awareness of physicians is indicated by the large number of WNF cases diagnosed in 2022 (36% of the total symptomatic cases) (Table 1). Moreover, as physicians are guided to send samples to the NRC and other specialized laboratories, which actively notify all diagnosed cases to the NPHO (active laboratory-based surveillance), the under-reporting of cases has been minimized. 

The prompt and thorough case investigation ensures the prompt identification of the most likely place of exposure of the patients; additionally, the timely response of the multi-sectoral “WG for the designation of areas affected by VBDs”, even in intense seasons, secures a quite timely and reliable identification of affected areas and a timely targeted implementation of response measures. 

Since 2010, a very limited number of transfusion-transmitted WNF infection (TT-WNV) events has been recorded, despite the high local notification rate of WNV infection in the general population [63]. The event of one case infected through blood transfusion in 2022 initiated a discussion regarding the reassessment of the criteria for the designation of WNV-affected areas and the triggers of the blood safety and hemovigilance measures. However, studies in Greece about WNV infection in blood donors and severe transfusion-transmitted WNV infection, during 2010–2021, have indicated the effectiveness of blood safety interventions, despite the high notification rate of WNV infection in the general population in several locations and seasons [63]. Details on the occurrence of positive blood donations and TT-WNV will be presented elsewhere.

## 5. Conclusions

WNV has become endemic in Greece, as well as in several neighboring and other European countries. Cases are expected to occur in each transmission season. Due to the complex epidemiology of the disease, the exact spatial and temporal distribution of cases cannot be predicted. Therefore, increased vigilance should be sustained in all related sectors for prompt detection of virus circulation in order to apply promptly the prevention and control measures. 

Moreover, the development of valid risk assessment and prediction tools, taking into account historical and current surveillance data, as well as climatic and environmental parameters that may influence virus circulation, would be of added value for the prompt implementation of prevention measures.

## Figures and Tables

**Table 1:** Epidemiological data of the annual seasonal outbreaks of West Nile virus (WNV) infection in Greece, 2010–2022 (total number of diagnosed cases of WNV infection = 1706), total number of neuroinvasive disease (WNND) cases = 1195).

Variable	2010	2011	2012	2013	2014	2015	2016	2017	2018	2019	2020	2021	2022
Number of WNV infection cases	262	100	161	86	15	0	0	48	317	227	145	59	286
Number of symptomatic WNV infection cases	262	100	161	86	15	0	0	48	311	224	142	57	278
Number of WNND cases	197	75	109	51	14	0	0	28	243	140	116	38	184
Percentage of WNND cases	75%	75%	68%	59%	93%	NA	NA	58%	77%	62%	80%	64%	64%
Notification rate of WNND cases (per 100,000 population)	1.8	0.7	1.0	0.5	0.1	0	0	0.3	2.2	1.3	1.1	0.4	1.7
Number of fatal cases with symptomatic WNV infection	35	9	18	11	6	0	0	5	51	35	23	8	33
Case fatality among cases with symptomatic WNV infection	13%	9%	11%	13%	40%	NA	NA	10%	16%	16%	16%	14%	12%
Number of fatal cases with WNND	33	9	18	10	6	0	0	5	48	33	23	7	33
Case fatality of cases with WNND	17%	12%	17%	20%	43%	NA	NA	18%	20%	24%	20%	18%	18%
Number of affected ^a^ municipalities	38	46	42	35	7	0	0	10	86	56	48	18	46
Number of affected ^a^ NUTS3 regional units	11	21	19	12	4	0	0	6	24	19	16	9	14
Number of affected ^a^ NUTS2 regions	5	7	8	5	3	0	0	3	7	5	4	4	5
Date of symptom onset of the first case	6/7	16/7	20/6	2/7	5/8	-	-	20/6	31/5	21/6	4/7	14/7	23/6

NA: not applicable; ^a^ municipalities, regional units, and regions with ≥1 case of WNV infection.

## Data Availability

Part of data presented in this study are openly available in the website of the National Public Health Organization (West Nile Virus Infection. Annual Epidemiological Reports for WNV Human Infection, Greece. 2010, 2011, 2012, 2013, 2014, 2017, 2018, 2019, 2020, 2021, 2022. Available online: https://eody.gov.gr/en/epidemiological-statistical-data/annual-epidemiological-data/ (accessed on 18 May 2023) [28]. Part of the data presented in this study are not publicly available and are available on request from the corresponding author.
